# Full Genome Characterisation of Bluetongue Virus Serotype 6 from the Netherlands 2008 and Comparison to Other Field and Vaccine Strains

**DOI:** 10.1371/journal.pone.0010323

**Published:** 2010-04-23

**Authors:** Sushila Maan, Narender S. Maan, Piet A. van Rijn, René G. P. van Gennip, Anna Sanders, Isabel M. Wright, Carrie Batten, Bernd Hoffmann, Michael Eschbaumer, Chris A. L. Oura, Abraham C. Potgieter, Kyriaki Nomikou, Peter P.C. Mertens

**Affiliations:** 1 Vector Borne Diseases Programme, Institute for Animal Health, Pirbright Laboratory, Woking, Surrey, United Kingdom; 2 Department of Virology, Central Veterinary Institute of Wageningen UR, AB Lelystad, The Netherlands; 3 Virology Division, Onderstepoort Veterinary Institute, Onderstepoort, South Africa; 4 Federal Research Institute for Animal Health, Greifswald-Insel Riems, Germany; Erasmus Medical Center, Netherlands

## Abstract

In mid September 2008, clinical signs of bluetongue (particularly coronitis) were observed in cows on three different farms in eastern Netherlands (Luttenberg, Heeten, and Barchem), two of which had been vaccinated with an inactivated BTV-8 vaccine (during May-June 2008). Bluetongue virus (BTV) infection was also detected on a fourth farm (Oldenzaal) in the same area while testing for export. BTV RNA was subsequently identified by real time RT-PCR targeting genome-segment (Seg-) 10, in blood samples from each farm. The virus was isolated from the Heeten sample (IAH “dsRNA virus reference collection” [dsRNA-VRC] isolate number NET2008/05) and typed as BTV-6 by RT-PCR targeting Seg-2. Sequencing confirmed the virus type, showing an identical Seg-2 sequence to that of the South African BTV-6 live-vaccine-strain. Although most of the other genome segments also showed very high levels of identity to the BTV-6 vaccine (99.7 to 100%), Seg-10 showed greatest identity (98.4%) to the BTV-2 vaccine (RSAvvv2/02), indicating that NET2008/05 had acquired a different Seg-10 by reassortment. Although Seg-7 from NET2008/05 was also most closely related to the BTV-6 vaccine (99.7/100% nt/aa identity), the Seg-7 sequence derived from the blood sample of the same animal (NET2008/06) was identical to that of the Netherlands BTV-8 (NET2006/04 and NET2007/01). This indicates that the blood contained two different Seg-7 sequences, one of which (from the BTV-6 vaccine) was selected during virus isolation in cell-culture. The predominance of the BTV-8 Seg-7 in the blood sample suggests that the virus was in the process of reassorting with the northern field strain of BTV-8. Two genome segments of the virus showed significant differences from the BTV-6 vaccine, indicating that they had been acquired by reassortment event with BTV-8, and another unknown parental-strain. However, the route by which BTV-6 and BTV-8 entered northern Europe was not established.

## Introduction

*Bluetongue virus* (BTV) is the ‘type’ species of the genus *Orbivirus*, within the family *Reoviridae* [1]. BTV is transmitted primarily by biting midges (*Culicoides spp*.) in which it also replicates, and is capable of infecting a wide range of ruminant, free ranging and captive species of wild ruminants [2], [3] as well as camelid species [4]–[7]. Some BTV strains have also been shown to be transmitted vertically, or via an oral route between individual mammalian hosts in the field [8]–[13], and after experimental infection [14], [15]. There are also recorded cases of large carnivores becoming infected after injection of BTV infected vaccines, or ingestion of infected meat [16]–[18].

Bluetongue (BT) is listed as a ‘notifiable disease’ by the Office International des Epizooties (OIE) [19], causing severe clinical signs that can include fever, lameness (coronitis), swelling of the head (particularly the lips and tongue) and death. The more severe forms of the disease are most frequently seen in sheep (particularly in the ‘improved’ mutton and wool breeds that are common in Europe) and in white-tailed deer (particularly in North America) [20]–[26]. Severe clinical signs and fatalities can occasionally also occur in cattle, goats and camelids [5], [27]–[29]. Although the infection is often inapparent in these other species, they can act as silent reservoirs, remaining viraemic for several months (particularly cattle) [25], [30]. However, even subclinical infection can carry significant costs, including loss of condition, reduced milk yield, infertility and abortion [31], as well as indirect costs associated with the export restrictions and the surveillance requirements imposed to limit the spread of the virus [32], [33].

Prior to 1998, occasional BT outbreaks had occurred in Europe, although in most cases these were relatively short lived (∼4–5 years) and involved a single BTV strain/serotype on each occasion (reviewed by Mellor et al. [34]). However, in 1998, a major series of BT incursions began in Europe, with new introductions in almost every subsequent year (to date), involving BTV-1, -2, -4, -6, -8, -9, -11 and -16 [35-38-www.reoviridae.org/dsRNA_virus_proteins/outbreaks.htm].

Live- BTV vaccines have been widely used to control the disease in the USA and as multivalent preparations (containing multiple serotypes) in Israel and southern Africa [39]–[41]. Monovalent ‘live-vaccines’ of BTV-2, 4, 8, 9 (western group) and BTV-16 (eastern group) have also been used, in attempts to minimise virus circulation in the Mediterranean region. Vector-borne spread of vaccine viruses has been reported before [42]–[44]. The release of these vaccine strains, some of which (including BTV-2 and 16) have persisted in the field, has further increased the level of genetic diversity within the European BTV population [45]–[47].

BTV incursions (from the east - possibly via Turkey and from North Africa) were initially restricted to Mediterranean Europe. However, during August 2006 sheep infected with a sub-Saharan African lineage of BTV-8, were identified in the Maastricht region of the Netherlands, the first time BTV had been detected in northern Europe [35]. This represented the start of the largest single outbreak of BT on record, subsequently spreading across the whole of Europe [35], [48], [49].

The BTV genome consists of 10 linear double-stranded RNA segments, which encode a total of 7 structural proteins (VP1 to VP7) and 3 distinct nonstructural proteins (NS1, NS2 and NS3/NS3a) [50]–[55]. The two inner layers of the BTV capsid (identified as the ‘sub-core’ and ‘core’) are composed of major structural proteins VP3 and VP7 (encoded by genome segments [Seg-] 3 and 7 respectively). The innermost subcore-shell surrounds the ten segments of the virus genome (one copy of each segment per particle) as well as three minor enzyme proteins VP1, VP4 and VP6 (encoded by Seg-1, 4 and 9 respectively). These core proteins and two of the non-structural proteins that are also synthesised in infected cells (NS1 and NS2 - encoded by Seg-5 and 8 respectively) are highly conserved and are antigenically cross-reactive between different strains of BTV. Although, the genome segments encoding these conserved proteins do show sequence-variations that reflect the geographic origin of the virus isolate (topotype), they show no significant correlation with the virus serotype [35], [53], [56]–[59]. Most BTV isolates that have been analysed can be divided into two major ‘eastern’ or ‘western’ topotypes, then into a number of further geographic subgroups, based on phylogenetic analysis of their nucleotide sequences [35], [56], [57], [60], [61].

Prior to 2008, 24 distinct serotypes of BTV had been identified, which can be distinguished in ‘neutralisation’ assays by the specificity of their reactions with the neutralising antibodies that are generated during infection of mammalian hosts. These neutralising antibodies interact with the more variable outer capsid proteins VP2 and VP5 (particularly VP2) encoded by Seg-2 and 6, respectively [1], [62]–[64]. Consequently Seg-2 and 6 vary in a manner that correlates with both the geographic origins (topotype) of the virus strain and virus serotype (particularly Seg-2), making them the most variable components of the BTV genome [35]. Different BTV ‘types’ can also be identified by conventional or real-time RT-PCR assays and/or sequence analyses targeting Seg-2 [35], [57]. Maan et al [57] showed that the Seg-2 sequencles of different BTV strains not only form distinct clades for each serotype, but also that the sequences of certain serotypes cluster more closely together, identifying ten distinct nucleotypes (identified as A–J).

In 2008 a virus was detected in goats from Switzerland (Toggenburg orbivirus - TOV), which shows a significant level of divergence in each of its genome segments, from the ‘major’ eastern and western topotypes of BTV. This virus has been provisionally identified (by serological and nucleic acid based analyses) as a novel 25^th^ BTV serotype (BTV-25) and represents a further nucleotype (K) [[65]–see discussion].

BTV Seg-7 and 10, which encode outer-core protein VP7 and NS3/NS3a respectively, show intermediate levels of nucleotide variation between different isolates, dividing them into a number of different clades that show only partial correlation with the geographic origin of the virus [35]. VP7 can mediate surface attachment, penetration and infection of insect cells by BTV cores [66], while NS3 has been associated with the release of virus particles from insect cells [67], [68], suggesting that these proteins may collectively influence the efficiency of BTV infection and dissemination within the vector insect. It has therefore also been suggested that the variations observed in Seg-7 (VP7) and Seg-10 (NS3) may relate to transmission of the virus by distinct insect vector species/populations in different geographical regions [35], [60], [69]–[71].

**Figure 1:**
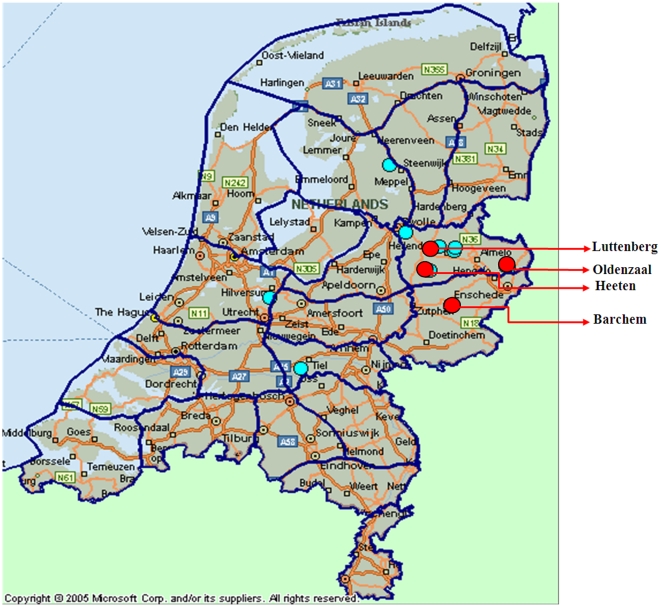
Map of geographical location of the BTV-6 affected farms in east of the Netherlands. The positions of the four farms originally sampled (Heeten, Barchem, Luttenberg and Oldenzaal) are indicated by red dots. Other farms where BTV-6 was detected are indicated by blue dots.

By 2008 western strains of BTV-1 and BTV-8 (originating in sub-Saharan Africa) had both become established in northern Europe [34], [72], [73]. However, despite vaccination campaigns, bluetongue infected animals were again identified during September 2008, on 4 farms in the east of the Netherlands (Heeten, Luttenberg, Barchem and Oldenzaal–Figure 1) and on November 5^th^ 2008 in Germany [74]. The virus was subsequently isolated from a bovine blood sample, taken from an animal showing clinical disease (Heeten), and was identified as BTV-6 (reported here). As part of attempts to clarify its origins, the complete nucleotide sequence of the virus genome was determined and compared to other European field strains, BTV vaccine strains used in the region, representative ‘eastern’ and ‘western’ BTV strains from other parts of the world, as well as the BTV-6 reference, vaccine and field strains from South Africa and USA.

## Methods

### Virus isolation and propagation in cell culture

EDTA treated blood samples from three cows (from farms at Barchem (10^th^ October 2008), Luttenberg (16^th^ October 2008) and Heeten (17^th^ October 2008) in eastern Netherlands), and one serum sample (Heeten), were sent to the Community Reference Laboratory (CRL) and Arbovirus Molecular Research Group (AMRG) at the Institute for Animal Health (IAH) Pirbright (21^st^ October 2008). These blood samples were taken from naturally infected animals in the field, by qualified veterinarians, as part of normal veterinary care and diagnostic testing procedures in the Netherlands. A sample of the blood from Heeten (stars number A163/08-3) is stored as sample ‘NET2008/06’ in the ‘dsRNA virus reference collection’ (dsRNA-VRC) at IAH Pirbright. dsRNA-VRC numbers for blood samples, or virus isolates use a generic format: ‘three letter country code, year of sampling/isolate number for that year’.

A 3.0 ml aliquot of the blood (NET2008/06) was washed three times with 10 ml of sterile phosphate-buffered saline (PBS). After each wash the red blood cells (RBC) were centrifuged at 3000×g for 5 min at 4°C and the supernatant was discarded. After the final wash, the RBCs were resuspended in 3.0 ml of PBS and RNA was extracted from 2.0 ml for RT-PCR and sequence analysis. One ml of the washed RBC was also used for virus isolation in *C. sonorensis* (KC) cells. At 7 dpi KC culture supernatant (KC1 - NET2008/04) was harvested and used to infect BHK-21 cells. The resulting virus isolate (KC1/BHK1 - NET2008/05) along with NET2008/04 and NET2008/06 were used for sequencing studies. Further details concerning the origins and passage history of individual virus isolates can be found at www.reoviridae.org/dsRNA_virus_proteins/ReoID/btv-6.htm.

### Serum neutralisation tests

Antiserum from the Heeten farm (sample number A163/08-2) from the same cow as virus isolate NET2008/05, was also sent to CRL at IAH Pirbright (21^st^ October 2008), for neutralising antibody testing against the 24 BTV serotypes in ‘serum neutralisation tests’ (SNT).

A standard ‘constant virus - varying serum’ method was used, with appropriate controls [75]. Briefly the test serum sample was heat inactivated at 56°C for ∼30 minutes, then serially diluted (1∶10). A fixed amount (100 VERO cell-TCID_50_/100 µl) of each of the 24 BTV serotypes was added to an equal volume of each serum dilution, in a 96 well plate (in four repeats), then incubated for ∼1 hour at 37°C followed by an overnight incubation at +4°C. This will allow antibodies to neutralise homologous virus. Fifty μl of a VERO cell suspension (2×10^5^ viable cells/ml), was then added to all wells, and the plate sealed with sterile cover then placed at ∼37°C (with 5% CO_2_). Plates were examined under a light microscope for cytopathic effects (CPE) at 3–4 days and 6–7 days post addition of cells. The neutralising antibody level was expressed as a ‘titre’ for each serotype, representing the inverse of the final dilution of serum at which each virus was neutralised, as calculated from the duplicate assays using the Karber formula [76]. Serum neutralising antibody titres of >10 were considered significant.

### Identification of BTV and molecular typing

Viral RNA for conventional and real time RT-PCR assays were extracted from cell-free infected tissue culture supernatants, and from EDTA treated blood, using the QIAamp Viral RNA Mini Kit (QIAGEN) as per manufacturer's protocol. RNA for synthesis of full-length cDNA copies of BTV genome segments was also purified from infected KC and from BHK cells using TRIzol reagent (Invitrogen) [57], [77], [78].

Serogroup-specific real-time RT-PCR assays targeting Seg-1 were carried out as described by Shaw et al. [79]. Conventional ‘type-specific’ RT-PCR assays (targeting Seg-2 of all 25 established BTV serotypes) were performed as described by Mertens et al. ([80] and–manuscript in preparation). Type specific real-time RT-PCR assays were carried out for the European BTV types, using assays kits supplied by (LSI–Laboratoire Service International), according to the manufacturer's instructions.

Terminal and internal primers that are specific for each genome segment were also used in conventional RT-PCRs, to amplify cDNAs for sequencing studies. These included type-specific and ‘nucleotype C’ specific primers targeting Seg-2 of BTV-6 (Table 1). In brief, a single-tube reaction contained the SuperScript™ III one-step RT-PCR system (Invitrogen) and high fidelity platinum® Taq was used with RNA templates extracted from blood or cell culture supernatant. The primer-template mix was heated to 95°C for 3 min to denature the viral dsRNA, followed by immediate cooling on ice, and addition of the reaction mix. Amplification of Seg-2, was carried out in 50 µL reaction volumes containing 25 µL of 2X reaction mix, 0.2 µM of each primer (1 µL of 10 µM,), 6 µL (1 pg–1 µg) of denatured RNA, 1 µL of SuperScript™ III RT/Platinum® Taq High Fidelity Enzyme Mix and nuclease free water to 50 µL. The RNA was reverse-transcribed at 55°C for 30 min. This was followed by an ‘inactivation/activation’ step at 94°C for 2 min (in order to simultaneously inactivate reverse transcriptase and activate the DNA polymerases), 40 amplification cycles (94°C for 15 sec, 55°C for 30 sec and 68°C for 1 min/kb), and a terminal extension step at 68°C for 5 min. The RT-PCR products were analysed and purified by 1% agarose gel electrophoresis (AGE) in tris acetate EDTA (TAE) buffer. The cDNA bands were stained with ethidium bromide, visualized under UV light, then excised and recovered from the gel for sequencing. Full length cDNA copies of individual BTV genome segments were also synthesised and amplified by RT-PCR, using the ‘anchor spacer–ligation’ method described by Maan et al. [35], [78] and Potgieter et al. [81].

**Table 1:** Primers for specific amplification of Seg-2 of BTV-6, by RT-PCR.

Primer pairs	Individual forward and reverse primers*	Primer Sequence (5′ to 3′)	Position on genome segment 2 (nt)	Predicted Product Size (bp)
Primer pair I	BTV-6/2/301F	GGTGGTATGTATAGAGGAAG	875-894	1631**
	BTV-6/2/790R	ACCACGCTACTCTGTATGCC	2506–2487	
Primer pair II	BTV-6/2/153F	CGAGGCGATTGGTGACACAGGT	461-482	2226
	BTV-6/2/853R	CAAAGGGAACCTCGCGCGTAATC	2687–2664	
Primer pair III	BTV-6/2/35F	GAGCGAAGATGATGAGGT	107–124	1211
	BTV-6/2/439R	GTCTTCTTCGTCTGTGAGATCAA	1318–1295	

*Individual primers are identified by the BTV serotype (e.g. BTV-6) followed by the number 2 (to indicate Seg-2), then a number to indicate the relative amino acid position of the primer within VP2, followed by F or R to indicate forward or reverse orientation.

**This primer pair also amplifies Seg-2 of BTV-14 and BTV-21, the most closely related serotypes to BTV-6 within nucleotype ‘C’ and it is therefore regarded as nucleotype ‘C’ specific.

The different cDNA amplicons were purified by AGE, recovered using a GFX™PCR DNA and gel band purification kit' (Amersham Pharmacia Biotech, Inc), then sequenced using an Applied Biosystems BigDye ddNTP capillary sequencer. Consensus sequences from each segment were assembled and analyzed using SeqMan Software (DNAStar Inc.).

The sequences generated were aligned with data for BTV genome segments from GenBank (Table S1) [35], [57], [72], using CLUSTAL W software [82]. Phylogenetic trees were constructed by neighbour-joining using distance matrices, generated by the p-distance determination algorithm in MEGA version 4.1 software (500 bootstrap replicates) [83]. Sequence relatedness is reported as percentage identity. The sequences obtained for each genome segment of NET2008/05 have been submitted to GenBank (accession numbers are shown in Table 2 and 3). Additional BTV-6 and BTV-8 field, reference and vaccine strains were also sequenced and submitted to Genbank (Table 3).

**Table 2:** Characteristics of dsRNA genome segments (cDNA copy) and proteins of the bluetongue virus serotype 6 Netherlands (NET2008/05).

†Genome segment (Size: bp)	ORFs (bp - including stop codon)	Protein nomenclature (§: protein structure/function)	Number of Amino acids (Da)	Location	Accession numbers	5′ Terminal sequences of the positive strand	3′ Terminal sequences of the positive strand
1 (39 44)	12-3920	VP1 (Pol)	1302 (149,476)	within the sub-core at the 5 fold axis	GQ506472	5′ - **GTTAAA** ATGCA**ATG**GTCGCA	**TGA** GAGCACGCGCCGCATTAC**ACTTAC** - 3′
2 (2922)	17-2885	VP2	955 (110,315)	outer capsid	GQ506473	5′- **GTTAAA**TTAGTTTCGTG**ATG**	TAA CCTCAGTGACATGGGGTTCACGAACTAATCA**ACTTAC** -3′
3 (2772)	17-2723	VP3 (T2)	901 (103,307)	sub-core capsid layer (T = 2 symmetry)	GQ506474	5′- **GTTAAA**TTTCCGTAGCC**ATG**	TAG ATGTGCGACCAATCTATGCACTTGGTAGCGGCAGCGGGAACAC**ACTTAC** -3′
4 (1981)	9-1943	VP4 (Cap)	644 (75,199)	within the sub-core at the 5 fold axis	GQ506475	5′- **GTTAAA**AC**ATG**CCTGAGCCA	TAA TGCGTGACTGCTAGGTGAGGGGGGCATGTGCA**ACTTAC** -3′
5 (1772)	35-1693	NS1 (TuP)	552 (64,445)	Cytoplasm forms tubules	GQ506476	5′- **GTTAAA**AAAGTTCTCTAGTTGGCAACCACCAAAC**ATG**	TAG TTACCGACTTCTGTTTTCTGTTTCTTCTTTTTTCTTTTTCTATTTTCTCTTAGCACTCTACTAGAACTTTTCA**ACTTAC** -3′
6 (1637)	28-1608	VP5	526 (59,199)	outer capsid	GQ506477	5′- **GTTAAA**AAGATCCCCATGATCGCGAAAG**ATG**	TGACAGCGGTGATCCCGGGACTTAC**ACTTAC**-3′
7 (1156)	18-1067	VP7 (T13)	349 (38,587)	Outer core (T = 13 symmetry)	GQ506478	5′- **GTTAAA**AATCTCTAGAG**ATG**	TAGTCCACTTTGCACGGGTGTGGGTTATGCGGGTGGTGTGTCGGTTGCAAGAAATATGTGTCTTGTTTAAACGTCTCTGGGATTAC**ACTTAC**-3′
8 (1125)	20-1084	NS2 (ViP)	354 (40,698)	Cytoplasm, viral inclusion bodies (VIB)	GQ506479	5′- **GTTAAA**AAATCCTTGAGTC**ATG**	TAGGCGCTTGTGACCGCGTGGTTAGGGGGGGATTTTAC**ACTTAC**-3′
9 (1049)	16-1005	VP6 (Hel)	329 (35,596)	Within the sub-core at the 5 fold axis	GQ506480	5′- **GTTAAA**AAATCGCAT**ATG**TC	TAAAGGGTCCAGGGTACCTTCTTGACGTAGGGCGATTTTAC**ACTTAC**-3′
10 (822)	20-709	NS3	229 (25,556)	Cell Membrane	GQ506481	5′- **GTTAAA**AAGTGTCGCTGCC**ATG**	TGAGGACAGTAGGTAGAGTGGCGCCCCGAGGTTTGCGTCGTGCAGGGTGGTTGACCTCGCGGCGTAGACTCCCACTGCTGTATAACGGGGGAGGGTGCGCGACACTACAC**ACTTAC**-3′
10 (822)	59-709	NS3a	216 (23,966)	Cell Membrane	GQ506481	5′ - **GTTAAA** AAGTGTCGCTGCCATGCTATCCGGGCTGATCCAAAGGTTCGAAGAAGAAAGG**ATG**	TGAGGACAGTAGGTAGAGTGGCGCCCCGAGGTTTACGTCGTGCAGGGTGGTTGACCTCGCGGCGTAGACTCCCACTGCTGTATAACGGGGGAGGGTGCGCGACACTACAC**ACTTAC**-3′

Pol =  RNA **pol**ymerase; Cap =  **cap**ping enzyme (guanylyltransferase); Hel  =  **hel**icase enzyme [42]; T2  =  protein with **T = 2** symmetry; T13  =  Protein with **T = 13** symmetry; ViP =  **v**iral **i**nclusion body matrix **p**rotein; TuP =  **tu**bule **p**rotein. Letters in bold blue are the 5′ and 3′ terminal conserved sequences in genome segments of BTV-6 NET2008/05.

**Table 3:** Accession numbers of BTV strains sequenced and used for phylogenetic analyses.

IAH Reference collection number or Year of isolation or strain designation	Country of origin	Seg-1	Seg-2	Seg-3	Seg-4	Seg-5 (NS1)	Seg-6 (VP5)	Seg-7	Seg-8	Seg-9	Seg-10
**BTV-6 and 8 full genomes sequenced in these studies**											
BTV-6 Vaccine strain	S. Africa	GQ506506	GQ506507	GQ506508	GQ506509	GQ506510	GQ506511	GQ506512	GQ506513	GQ506514	GQ506515
BTV-6 NET2008/05	Netherlands	GQ506472	GQ506473	GQ506474	GQ506475	GQ506476	GQ506477	GQ506478	GQ506479	GQ506480	GQ506481
BTV-6 Underberg S. Africa (P635- isolated from midges)	S. Africa	GQ506488	GQ506489	GQ506490	GQ506491	GQ506492	GQ506493	GQ506494	GQ506495	GQ506496	GQ506497
BTV-6 Prototype vaccine strain (5011-60E)	S. Africa	GQ506526	GQ506527	GQ506528	GQ506529	GQ506530	GQ506531	GQ506532	GQ506533	GQ506534	GQ506535
BTV-6 RSArrrr/06	S. Africa	GQ506498	AJ585127	GQ506499	GQ506500	GQ506501	AJ586703	GQ506502	GQ506503	GQ506504	GQ506505
BTV-6 S. Africa (BT-2-03 - isolated from a lamb)	S. Africa	GQ506516	GQ506517	GQ506518	GQ506519	GQ506520	GQ506521	GQ506522	GQ506523	GQ506524	GQ506525
BTV-6 USA2006/01	USA	GQ506536	GQ506537	GQ506538	GQ506539	GQ506540	GQ506541	GQ506542	GQ506543	GQ506544	GQ506545
BTV-8 NET2007/01	Netherlands	GQ506451	GQ506452	GQ506453	GQ506454	GQ506455	GQ506456	GQ506457	GQ506458	GQ506459	GQ506460
**BTV-6 partial genomes sequenced in these studies**											
BTV-6 GER2008/05	Germany	x	GQ506461	x	GQ506462	GQ506463	GQ506464	GQ506465	GQ506466	GQ506467	x
BTV-6 NET2008/04	Netherlands	GQ506468	GU550956	GQ506469	GQ506470	x	x	GQ506471	x	x	x
BTV-6 NET2008/06	Netherlands	x	GU550957	x	x	GQ506482	GQ506483	GQ506484	GQ506485	GQ506486	GQ506487
**Published partial genome used in these studies**											
BTV-25 (Toggenburg Orbivirus [TOV])	Switzerland	GQ982522	EU839840	GQ982523	GQ982524	EU839841	EU839842	EU839843	EU839844	EU839845	EU839846

## Results

### Identification of BTV in the Netherlands

Blood samples were taken during October 2008, from three cows showing mild clinical signs, consistent with BTV infection, from different farms in the eastern Netherlands (at Heeten, Luttenberg and Barchem). Two if these animals (Heeten and Barchem) had been vaccinated against BTV-8 during May/June 2008. A fourth apparently healthy cow was also sampled as part of routine pre-export testing from a farm at Oldenzaal. These samples tested positive for the presence of BTV RNA by RT-PCR (targeting Seg-10). Partial sequence analyses of Seg-10 showed that although one of the samples (Luttenberg) contained a “mixed sequence” (and was therefore unreadable), the other three samples (from Heeten, Barchem and Oldenzaal) showed significant sequence differences when compared to the previous northern European outbreak strain of BTV-8 (∼95% identity over a 200 base pair region [35]) and BTV-1 (82.0–83.3% identity over full length region). These PCR results were confirmed by re-sampling and re-testing of the RT-PCR positive animals.

### Virus isolation and propagation in cell culture

A sample of blood taken from a cow in eastern Netherlands (Heeten) (sample number A163/08-3) in October 2008, which had tested positive for bluetongue in a Seg-10 based real time RT-PCR assay conducted at the Central Veterinary Institute of Wageningen UR (CVI), Lelystad, was sent to the CRL at IAH Pirbright in October 2008 (stored as dsRNA-VRC sample number NET2008/06). The virus was isolated (single passage) in KC cells without CPE (generating isolate NET2008/04) then passaged in BHK-21 cells (isolate NET2008/05), causing 100% CPE at 4 days post infection. Subsequent real-time RT-PCR assays (at IAH) targeting BTV Seg-1 [79], gave CT values of 26 for the Heeten blood sample (NET2008/06), and 12–35 for cell-culture supernatants from isolates NET2008/05 and NET2008/04, confirming the presence of BTV.

### Identification and typing of BTV-6

Conventional RT-PCR assays (at CVI) using primers directed against Seg-2 of the BTV serotypes recently detected in Europe (BTV-1, 2, 4, 8, 9 and 16 [80] see: www.reoviridae.org/dsRNA_virus_proteins/ReoID/BTV-S2-Primers-Eurotypes.htm) failed to amplify any cDNA products of the appropriate sizes from the four bovine blood samples previously identified as positive by BTV group-specific RT-PCR. This indicated that the new virus represented an additional European serotype. However, subsequent RT-PCR assays (at IAH) using two sets of experimental primers targeting Seg-2 from each of the BTV serotypes (1 to 25) (Mertens et al., [80] and manuscript in preparation), generated cDNA amplicons of the expected sizes from two of the three blood samples (Heeten and Barchem), but only with the BTV-6 specific primers. These analyses not only identified the virus as BTV-6, but also excluded the other 24 BTV serotypes, providing the first positive identification of this type anywhere in Europe. However, one of the blood samples (Luttenberg) from an unvaccinated cow gave positive results with both the BTV-6 and BTV-8 specific primers, indicating that this animal had recently been infected with both serotypes.

**Figure 2:**
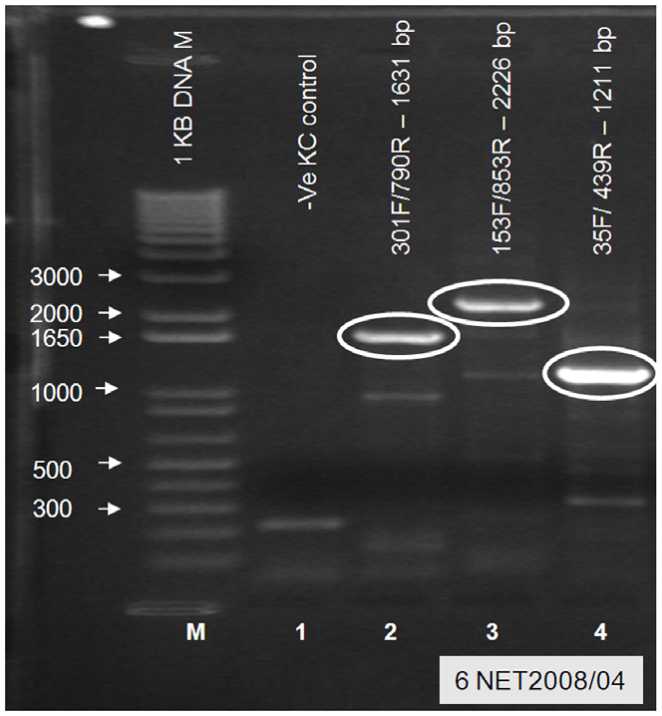
Electrophoretic analysis of cDNA products generated from Seg-2 of NET2008/04 using nucleotype ‘C’ and BTV-6 specific primer pairs. PCR amplicons were generated from cDNA of BTV-6 isolate NET2008/04 with one nucleotype ‘C’ (lane 2) and two type 6 specific primer pairs (lanes 3 and 4 – Table 1). No specific amplification was seen with these primers from mock KC cells (Lane 1). Lane M: 1 kb marker.

RT-PCRs using two further pairs of BTV-6 specific primers and one pair of primers specific for ‘nucleotype C’ (which includes BTV-6, 14 and 21) (Table 1) were used to generate cDNAs of the expected sizes from virus isolates NET2008/04 and NET2008/05 (derived from the Heeten blood sample), representing a total of ∼75% of Seg-2. This confirmed the identification of BTV-6 and provided cDNAs for sequence analyses (Figure 2). Type-specific real-time RT-PCR assays (supplied by LSI), targeting Seg-2 of BTV-1, 2, 4, 6, 8, 9, 11, 16 and 25, also subsequently generated positive results for BTV-6 from these isolates (CT value of 38 [NET2008/04] and 14 [NET2008/05]), as well as from the original Heeten blood sample (CT value of 27 [NET2008/06]).

### Typing of BTV specific antibodies in sera from the Netherlands

Antiserum from the BTV-8 vaccinated cow from the Heeten farm (sample number A163/08-2), was also tested using serum neutralisation tests (SNT) against reference strains of BTV serotypes 1 to 24, and gave high neutralisation titres against both BTV-6 and BTV-8 (in each case at a neutralisation titre of 1/1280). Although, lower titres were also detected against two other serotypes (1/80 [BTV-14] to 1/120 [BTV-18]), these results are consistent with sequential infection by BTV-6 and either infection or vaccination against BTV-8 [84], [85].

### Full-length sequence analyses and comparison of the NET2008/05 genome

In an attempt to identify the origins of the BTV-6 strain from Heeten, cDNAs were amplified and used to generate full length sequence data for individual genome segments of the virus (segments 1, 3, 4, 7 were sequenced from KC cell (KC1) isolate, NET2008/04; while the whole genome was sequenced from the KC1/BHK1 isolate, NET2008/05, and segments 2 (partial), 5 to 10 were sequenced from the Heeten blood sample NET2008/06). The consensus sequences obtained for most of the genome segments were identical in each case, except for Seg-7 and Seg-10 (see below). The remainder of the paper therefore refers to the sequence of the Netherlands BTV-6 KC1/BHK1 isolate (NET2008/05), except where specific changes were detected between the different samples.

#### Genome segment 1

Seg-1 of BTV-6 NET2008/05 was compared to other ‘eastern’ and ‘western’ BTVs, including other strains of BTV-6 sequenced in this study (Table 2 & 3). In each case Seg-1 is 3944 base pairs (bp) long, encoding the 1302 amino acid (aa) RNA dependent RNA polymerase (RdRp - Pol) (Table 2), and is highly ‘conserved’, showing >78.7% overall identity. Seg-1 of BTV-6 NET2008/05 was most closely related to the South African BTV-6 vaccine strain (5011-60E-VP1) (99.9% nucleotide [nt] identity), a BTV-6 strain from Germany (GER2008/05) (99.9% identity), and the BTV-6 reference strain (RSArrrr/06–99.8%).

**Figure 3:**
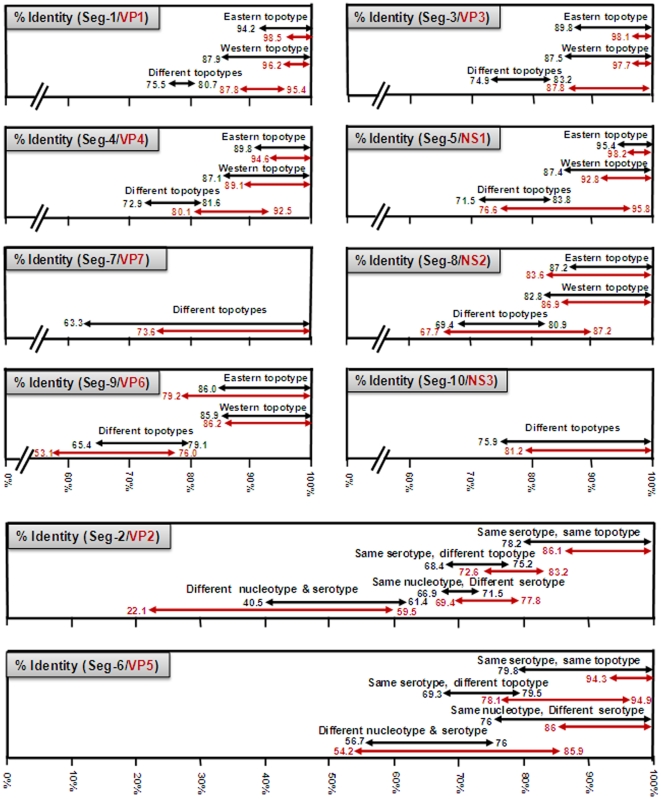
A graphical representation of the levels of nucleotide and amino acid sequence identities detected in different genome segments and proteins, within and between different BTV serotypes and topotypes. Estimates of the levels of identity in each genome segment/protein, between different BTV strains are based on multiple datasets for widely distributed isolates, as described previously by Maan et al. [35], [57], [72] and listed in tables 3 and S1. The values presented here show the levels of identity detected within the ‘major’ eastern and western topotypes for each genome segment (black) and protein (red). The levels of identity that are shown between different topotypes, include data for all genome segments of BTV-25 (Toggenburg orbivirus), as well as Seg-3 of BTV-15 Australia (Ac. No. AY322427) and Seg-5 of BTV-20 Australia (Ac. No. X56735), as representatives of distinct topotypes.

The deduced aa sequence for BTV VP1 is also highly conserved, showing >93.2% identity, with a clear separation between ‘eastern’ and ‘western’ virus groups/topotypes (with a maximum of 5.8/1.7% and 12.0/3.8% nt/aa intra-topotype variation respectively within these two groups) (Figure 3) [35]. The level of nt/aa identity in Seg-1/VP1 between these major eastern and western virus groups was 78.7 to 80.7/93.2 to 95.4% respectively (Figure 3). However, BTV-25 (TOV) does not cluster with either group, showing <75.8/88.1% and <75.8/88.2% nt/aa identity with the eastern or western BTV strains respectively, suggesting that it represents a further distinct (western) group/topotype.

Seg1/VP1 from the BTV-6 Netherland isolates was identical to that of the BTV-6 vaccine strain, with 99.8% identity to the reference strain (RSArrrr/06) from which the vaccine was originally derived [39]. Seg-1/VP1 of BTV-6 NET2008/05 was also closely related to those of other BTV-6 strains from South Africa (95.1 to 95.2% nt and 98.6 to 98.8% aa identity), but somewhat less closely related to the western strain of BTV-6 from the USA (USA2006/01–88.9/98.4% nt/aa identity).

#### Genome segment 2

**Figure 4:**
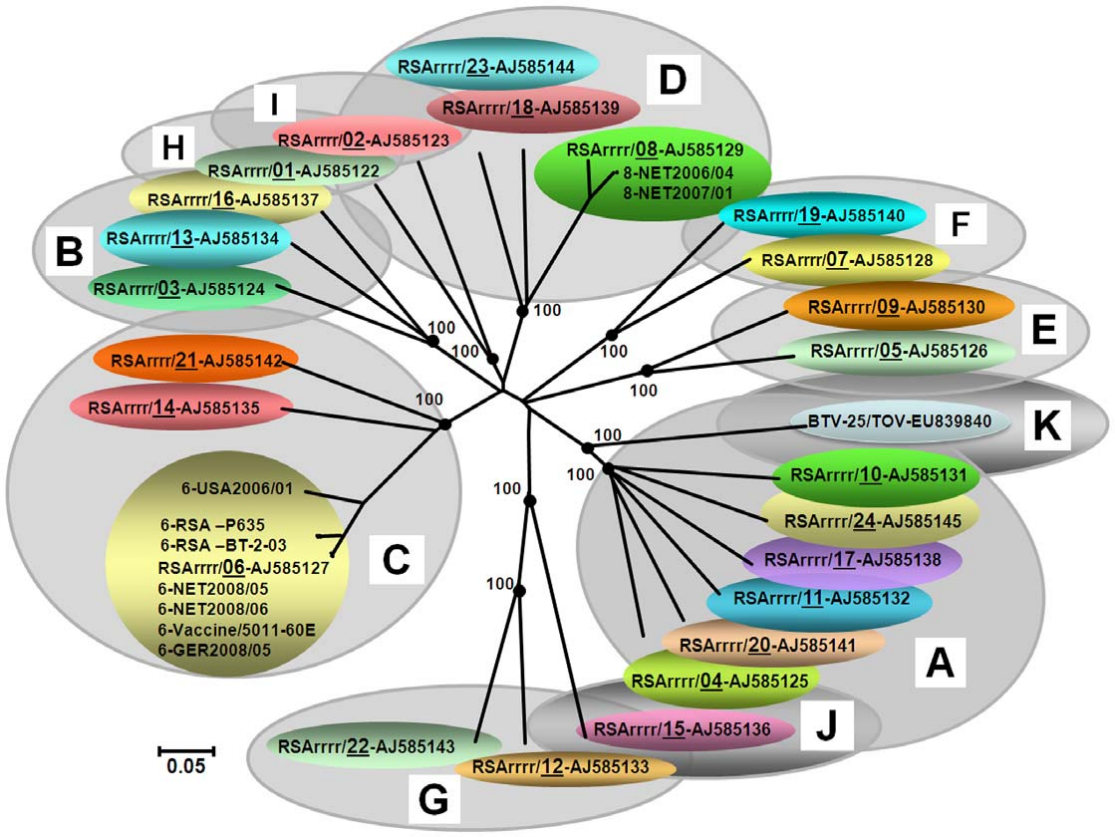
Neighbour-joining tree showing relationships between Seg-2 of NET2008/05 with other BTV-6 isolates and the twenty-five reference strains of different BTV serotypes. The tree was constructed using distance matrices, generated using the p-distance determination algorithm in MEGA 4.1 (500 bootstrap replicates) [83]. The ten evolutionary branching points are indicated by black dots on the tree (along with their bootstrap values), which correlate with the eleven ‘Seg-2 nucleotypes’ designated A–K. BTV-25 (Toggenburg orbivirus [TOV]) forms a new 11^th^ Seg-2 nucleotype (K). Members of the same Seg-2 nucleotype, are characterised by >66.9% identity in their Seg-2 nucleotide sequences, while members of different nucleotypes show <61.4% identity in Seg-2 [57], [72] (see Figure 3). The trees shown in Figures 4-7 were drawn using same parameters.

Multiple cDNAs were amplified from Seg-2 of NET2008/05 and sequenced (full–length), showing that like other BTV-6 strains, it is conserved at 2922 bp, encoding VP2, which is 955 aa long (Table 2). Comparisons with Seg-2/VP2 from the reference strains of BTV 1 to 25 (Table 3) [57], [65], [72], showed that the Netherlands strain of BTV-6 groups within ‘Seg-2 nucleotype C’ along with reference strains of BTV-6, 14 and 21 (Figure 3 and 4). It is very closely related to the reference strain of BTV-6 (99.8/99.7% nt/aa identity) and is identical to the vaccine strain of the same serotype from South Africa (Figure 4). This analysis not only confirmed its initial identification of BTV-6, but also demonstrates that NET2008/05 was derived from the ‘vaccine’ strain.

Comparisons to sequence data for Seg-2 from multiple subsequent isolates of BTV-6 from northern Europe (2008-2009 - including GER2008/05) showed that they all group closely with NET2008/05 (>99.9/100% nt/aa identity)–indicating a common origin, with only 89.1/95% nt/aa identity to BTV-6 strain from USA (USA2006/01). Field isolates of BTV-6 from South Africa (Table 3) were also closely related to each other (with 99.3/99.5% nt/aa identity) but more distantly related to NET2008/05 (95.7 to 95.9% nt and 98.5 to 98.6% aa identity) or USA2006/01 (88.6 to 88.7% nt and 94.7 to 94.8% aa identity–Figure 4). No BTV-6 strains belonging to the major eastern topotypes/origins were available for comparison.

#### Genome segment 3

Seg-3 of the different European BTV strains is 2772 bp long (regardless of serotype or topotype), encoding the 901 aa of the highly conserved BTV sub-core-shell protein, VP3(T2) (Table 2). The eastern and western virus isolates formed two distinct clusters (major topotypes), which have nt/aa identities of >89.80/98.1% (eastern topotype) and 87.5/97.7% (western topotype) respectively. The overall level of nt/aa identity in Seg-3/VP3 between these eastern and western virus groups, was 79.3 to 82.4/96.9 to 99.3% respectively (Figure 3). However a single Australian strain of BTV-15 (Ac. No. AY322427) appears to represent a further distinct ‘far eastern’ group, showing <80.8/97.9% nt/aa identity with the other eastern strains and 78.9 to 82.4/95.4 to 97.6% nt/aa identity with western strains. In a similar manner, BTV-25 (TOV) shows <77/88.9% and <76.6/89.5% nt/aa identity, respectively, with the major eastern and western topotypes of BTV, again indicating (as with Seg-1/VP1) that it represents a further distinct (western) group/topotype.

Seg-3/VP3 of BTV-6 NET2008/05 showed >88.5% nt identity to the other European viruses within the major ‘western’ topotype, but only 79.8 to 83.2% identity to the ‘eastern topotype’. Within the western group Seg-3/VP3 of NET2008/05 was most closely related to the BTV-6 vaccine and reference strains (99.8/99.9% nt/aa identity), and more distantly related to South African field isolates of BTV-6 (94.6 to 94.7/99.4 to 99.6% nt/aa identity), or the North American BTV-6 (USA2006/01 - 90.1/97.2% nt/aa identity). The Netherlands virus has a unique aa change in VP3, when compared to the BTV-6 vaccine or reference strains, at position 266 (Valine to isoleucine).

#### Genome segment 4

Seg-4 of BTV is consistently 1981 nt in length, encoding the 644 aa of the highly conserved BTV capping and transmethylase enzyme - VP4(CaP) (Table 2). The full-length sequence of Seg-4/VP4 from BTV-6 NET2008/05 grouped with other ‘western’ BTV strains (showing >89.6/95.3% nt/aa identity), but only 79.4 to 79.9/90.4 to 91.8% nt/aa identity to the ‘eastern’ isolates.

Analysis of Seg-4/VP4 sequences again divided the majority of the isolates compared, into either an eastern or a western topotype, with nt/aa identities of >89.8/94.6% (eastern topotype) and 87.1/89.1% (western topotype) respectively. The level of nt/aa identity between these major topotypes was 77.7 to 81.6/86.1 to 92.5% (Figure 3). However, BTV-25 (TOV) showed <73.4/82.1% and <73.4/82% nt/aa identity with the eastern and western BTV strains respectively, which is consistent with its membership of a further distinct (western) group/topotype.

Seg-4/VP4 of BTV-6 NET2008/05 show 99.9/100% and 99.8/100%, nt/aa identity, to the South African vaccine and reference strains of type 6 (respectively), indicating a common origin. Like several of the other genome segments, Seg-4 of BTV-6 NET2008/05 also showed a slightly more distant relation with the BTV-6 field strains from South Africa (93.7–93.8/98% nt/aa identity), and the American BTV-6 (USA2006/01 - 90.1/97.2% nt/aa identity). Four unique aa changes were detected in the Netherlands and vaccine or reference strains of type 6, as compared to other western BTV isolates (at positions 54 (V-I), 117 (F-Y), 166 (I-T) and 825 (I-V)).

#### Genome segment 5

Seg-5 of BTV- 6 NET2008/05 is 1772 nt long, encoding the 552 aa of the NS1 tubule protein (TuP), with upstream and downstream NCRs of 34 and 81 bp in length (Table 2). The downstream NCR of BTV Seg-5 varies in length, even between isolates of the same serotype [35]. Seg-5 of the BTV-6 isolates from the Netherlands and South Africa has an 85 bp long 3′ NCR, while that BTV-6 from the USA (USA2006/01) is only 78 bp.

Seg-5/NS1 from the majority of the different BTV isolates compared also segregate into two ‘major’ groups, with nt/aa identities of >95.4/98.2% (eastern topotype) and >87.4/92.8% (western topotype) respectively (Figure 3). The overall level of nt/aa identity between these major topotypes was 81.6 to 83.8/92.4 to 95.8%.

However a single Australian strain of BTV-20 (Ac. No. X56735) appears to represent a further distinct eastern group, showing <81.5/92.4% nt/aa identity with the other eastern strains and 78.9 to 80.9/89.7 to 91.7% nt/aa identity with western strains. BTV-25 (TOV) shows <74.3/78.1% and <74.0/78.6% nt/aa identity with the eastern and western strains of BTV respectively, again suggesting that it represent a further distinct (western) group/topotype.

Seg-5/NS1 of BTV-6 NET2008/05 belong to the major western topotype, showing 100% identity with the South African BTV-6 vaccine and reference strains (again indicating a common origin), with 92.9/99.3% and 88.2/96.7% nt/aa identity to other BTV-6 strains from South Africa and USA respectively. Seg-5/NS1 of BTV-6 NET2008/05 also showed 98.8%, 98.6%, and 98.1% nt identity, to south African vaccine strains of BTV-1, 9 and 4 respectively, and up to 94.2/100% nt/aa identity to BTV-8 strains from Europe.

#### Genome segment 6

**Figure 5 pone-0010323-g005:**
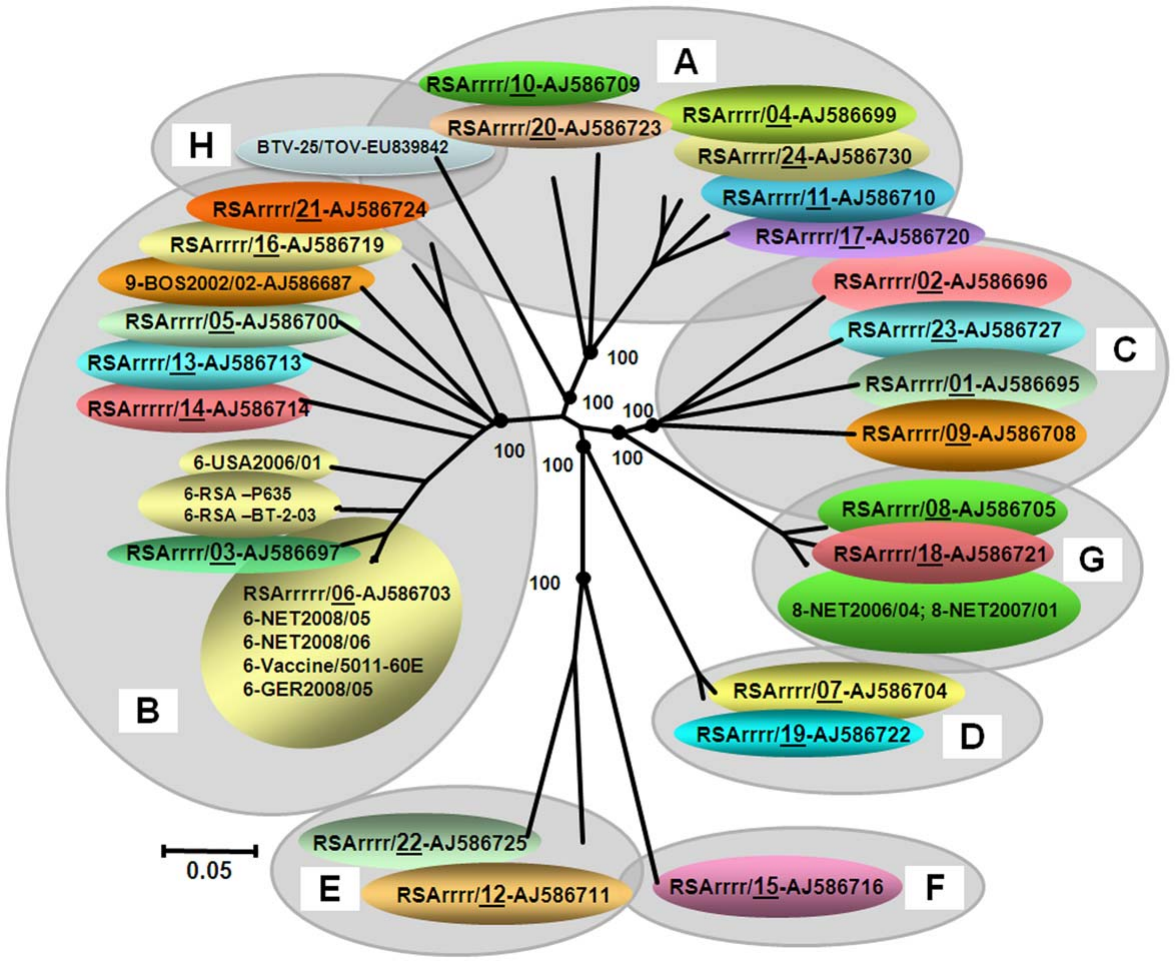
Neighbour-joining tree showing relationships between Seg-6 of NET2008/05 with other BTV-6 isolates and the twenty-five reference strains of different BTV serotypes. The seven evolutionary branching points are indicated by black dots on the tree (along with their bootstrap values), dividing the sequences into eight ‘Seg-6 nucleotypes’ designated ‘A–H’. In previous studies [72], six Seg-6 nucleotypes were identified, however the additional analyses described here indicate that the previous ‘nucleotype C’ should be subdivided (into nucleotypes ‘C’ and ‘G’). BTV-25 (Toggenburg orbivirus [TOV]) forms a new 8^th^ Seg-6 nucleotype (H). Members of the same nucleotype show >76% nt identity in Seg-6, while members of different nucleotypes show <76% nt identity (see Figure 3).

Seg-6 encodes VP5, the smaller of the two outer capsid components and second most variable of the BTV proteins. Although variations in Seg-6/VP5 can also be used to identify eight nucleotypes (A-H - Figure 5, see discussion[65], [72]), unlike Seg-2/VP2, they show only partial correlation with BTV serotype. This is demonstrated by the close relationships between BTV-7 and 19 within nucleotype D, and the more distant relationship between the reference strain of BTV-9 in nucleotype C, and the Bosnian strain of BTV-9 in nucleotype B (Figure 5). The levels of nt/aa identity in Seg-6/VP5 between different serotypes that are also present in different nucleotypes are 56.7 to 76/54.2 and 85.9% respectively. However different serotypes within the same nucleotype can show very high levels of identity approaching 100% (Figure 3). Seg-6 of BTV types 1, 2, 9 and 16 all show evidence for separation into eastern and western topotypes, [35], [72]. In contrast the BTV-4, -6 and -8 strains that have been analysed are exclusively from western origins, and consequently there is (as yet) no evidence for separation of their Seg-6 sequences into eastern and western topotypes.

Seg-6/VP5 of the BTV-6 isolates analysed (Table 2 and 3), are conserved at 1637 bp/526 aa long, with >89.3/98.5% nt/aa identity overall. Seg-6 of NET2008/05 grouping within ‘nucleotype B’, with reference strain of BTV-3 from South Africa (RSArrrr/03–nt/aa identity of 96.1 to 96.5/99.6%) and BTV-6 from the USA (USA2006/01–nt/aa identity of 88.9 to 89.3/98.7%) (Figure 5). The majority of these BTV-6 isolates (with the exception of BTV-6 USA2006/01) belong to an African sub-group, with >94.6/99.4% nt/aa identity. However, the European strains (represented by NET2008/05) were even more closely related to the reference strain of BTV-6 (RSArrrr/06) with only 5 nt and 2 aa differences (99.7/99.6% nt/aa identity) and are identical to the BTV-6 vaccine strain (RSAvvvv/06). This confirms the initial identification of the northern European isolate as type 6 that was made by Seg-2 RT-PCR. Subsequent analyses of multiple northern European isolates of BTV-6 (including GER2008/05) show that they all contain Seg-6 that is identical to that of NET2008/05, and therefore all appear likely to be derived from the same origin.

Recent South African isolates of BTV-6 (Table 3) are closely related to each other, with >99.7/100% nt/aa identity, but are more distantly related to NET2008/05 (with only 94.6 to 94.8/99.4% nt/aa identity), or BTV-6 from USA (USA2006/01) (89.5 to 89.6/98.5% nt/aa identity) (Figure 5), indicating that they do not share a very recent common ancestry with the northern European isolate.

#### Genome segment 7

**Figure 6 pone-0010323-g006:**
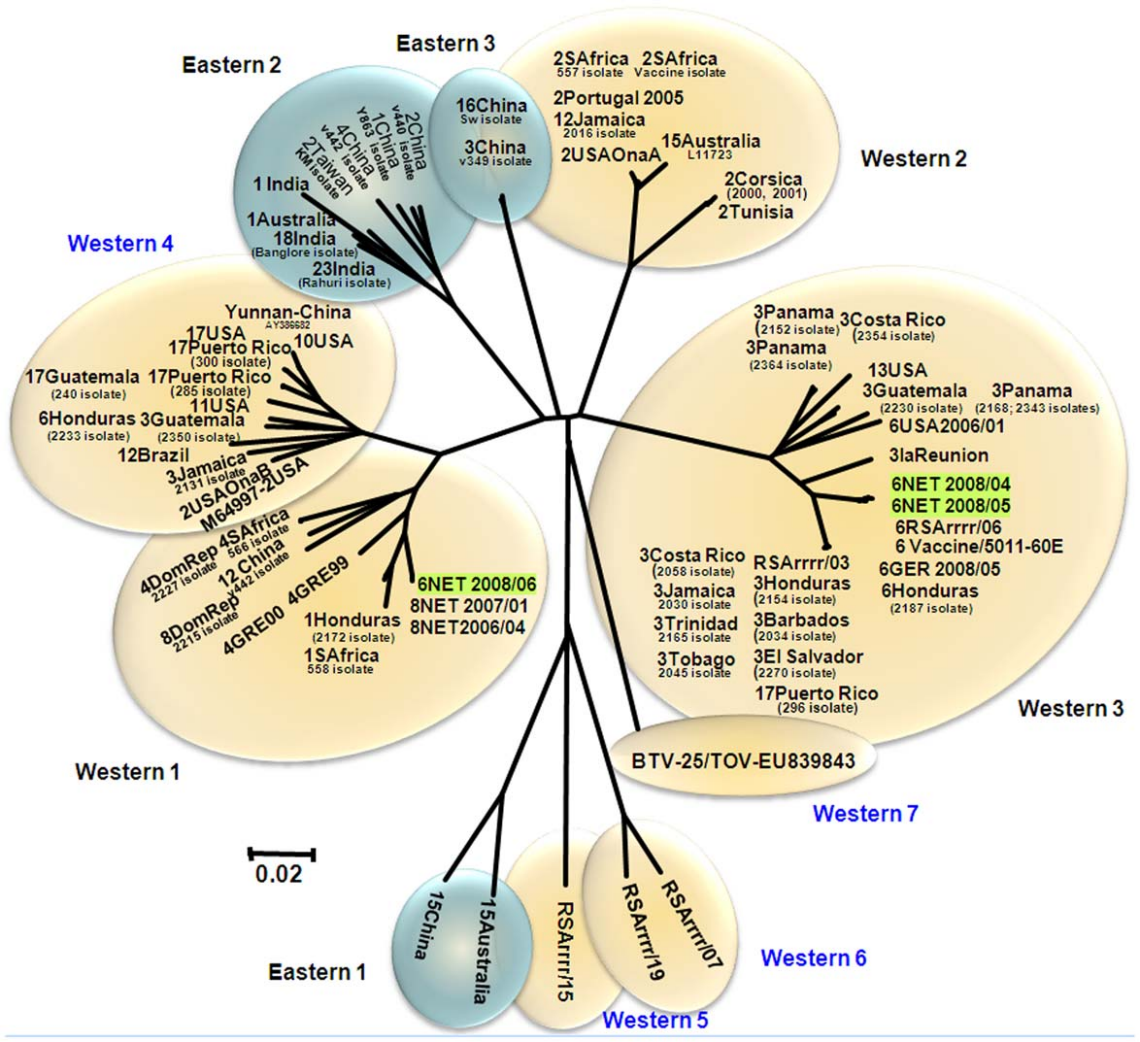
Neighbour-joining tree showing relationships between Seg-7 of NET2008/05 with other BTV-6 isolates and other BTV strains from different serotypes from around the world.

Seg-7 of NET2008/05 is 1156 bp long, encoding 349 aa of the major BTV serogroup-specific antigen and core surface protein - VP7 (Table 2). The aa sequence of VP7 is significantly more conserved (>73.6% identity) than the nt sequence (>63.3%) reflecting large numbers of synonymous mutations in the third base position (Figure 3) [35]. Seg-7 was reported to form six distinct clusters: three of these are primarily from western origins (western 1, 2 and 3) and three from an eastern origin (eastern 1, 2 and 3) [35]. However, Seg-7 from the Chinese strain of BTV-12 groups within western group 1, suggesting some movement of strains between geographic regions. Analysis of additional isolates from around the world also identified four additional western clusters, as well as an isolate from Yunnan, China (AY386682) in western group 4, and BTV-15 from Australia (Ac. No. L11723) within western group 2 (Figure 6). BTV-25 (TOV), showed 70 to 79.1%/79.9 to 93.4% nt/aa identity with other BTV strains, representing a further distinct group/topotype and forms a western group 7.

The nt/aa sequence of Seg-7/VP7 of NET2008/04 (KC isolate) and NET2008/05 (KC1/BHK1 isolate) showed >70.5/99.7% nt/aa identity to other BTV strains, and as observed with the majority of other segments (except Seg-10), highest levels of identity to the BTV-6 vaccine strains in ‘western cluster 3’. However, in contrast Seg-7 from NET2008/06 showed 79.3 to 79.9/94.5 to 94.8% nt/aa identity with other BTV-6 strains, but 100% nt/aa identity with BTV-8 from the Netherlands 2006/2007 (NET2006/04 and NET2007/01), within ‘western group 1’ (Figure 6).

#### Genome segment 8

Seg-8 of BTV is conserved at 1125 bp, encoding the 354 aa of the viral inclusion body (VIB) matrix protein - NS2 (Table 2). Seg-8 of the BTV isolates also showed clear separation into major eastern and western topotypes, with NET2008/05 grouping along with other western viruses, including the vaccine strains of BTV-2, 4 and 9 and European field strains of BTV-2 and 4 (data not shown). As observed with the other conserved segments, BTV-25 (TOV) forms a second distinct western topotype.

The BTV isolates that were compared from eastern origins, all showed nt/aa identities in Seg-8/NS2, of >87.2/83.6%, while the ‘western’ viruses showed >82.8/86.9% identity (Figure 3). The only exception was BTV-25 (TOV), which although showed <70.4/70.1% nt/aa identity with eastern isolates, only <71.1/69.5% nt/aa identity with the other western BTV strains. Overall, nt/aa identity levels of 75.0 to 80.5/80.0 to 87.2% were detected between the ‘major’ eastern and western topotypes of BTV (Figure 3). The results obtained, like those for the other conserved genome segments are consistent with BTV-25 (TOV), as a member of a further distinct (western) topotype (Figure 3).

Seg-8 of NET2008/05 and NET2008/06 showed >99.9% nt and aa identity to the vaccine strains of BTV-6 and to other BTV-6 isolates from Germany (GER2008/05). Although clearly distinct, the Netherlands strain also showed relatively high levels of nt/aa identity to South African field strains of BTV-6 (96/98%), and to BTV-6 from USA (USA2006/01–88.8/93.7%).

#### Genome segment 9

Seg-9 codes for VP6, a minor core protein and the helicase enzyme (Hel) of BTV. As in previous studies [35], the BTV Seg-9 nt sequences analysed here were clearly divided into eastern and western topotypes. Seg-9 of the eastern BTV isolates is 1052 bp, encoding a protein 330 aa in length, while Seg-9 from the western lineage is 1049 bp (329 aa) (Table 2) [35]. Of the 112 BTV strains analysed, 96 isolates had two in frame initiation codons, at nucleotides 16 to 18 and 28 to 30 of Seg-9, which can generate two related forms of VP6. This agrees with previous observations of two closely migrating VP6 bands in purified BTV particles or in *in vitro* translation studies [86]. Only 16 isolates (belonging to both eastern and western topotypes) had a single start codon at nucleotides 28 to 30.

The level of nt/aa identity in Seg-9/VP6 within the ‘major’ eastern and western topotypes was >86.0/79.2% (eastern topotype) and >85.9/86.2% (western topotype) respectively, with 69.1 to 79.1/57.3 to 76.0% nt/aa identities between these groups (Figure 3). However, BTV-25 (TOV) showed <71.4/66.2% nt/aa identity with other western isolates; <71.8/66.6% with most eastern BTV isolates, but only (65.4/53.1% nt/aa) to BTV-15 Australia (Ac. No. DQ289044). As with the other genome segments analysed, these data suggest that BTV-15 Australia and BTV-25 (TOV) represent further distinct ‘far eastern’ and western BTV topotypes respectively.

Seg-9 of NET2008/05 belongs to the major western topotype and is identical to that of the South African BTV-6 vaccine, but shows minor differences (99.7/99.4% nt/aa identity) when compared to Seg-9/VP6 of the reference strain, from which the vaccine strain was derived. It is also closely related to but distinct from BTV-6 strains from South Africa (94.4 to 95.3/93.6 to 94.8% nt/aa identity), and the USA (USA2006/01 - 90.0/91.0% nt/aa identity).

#### Genome segment 10

**Figure 7:**
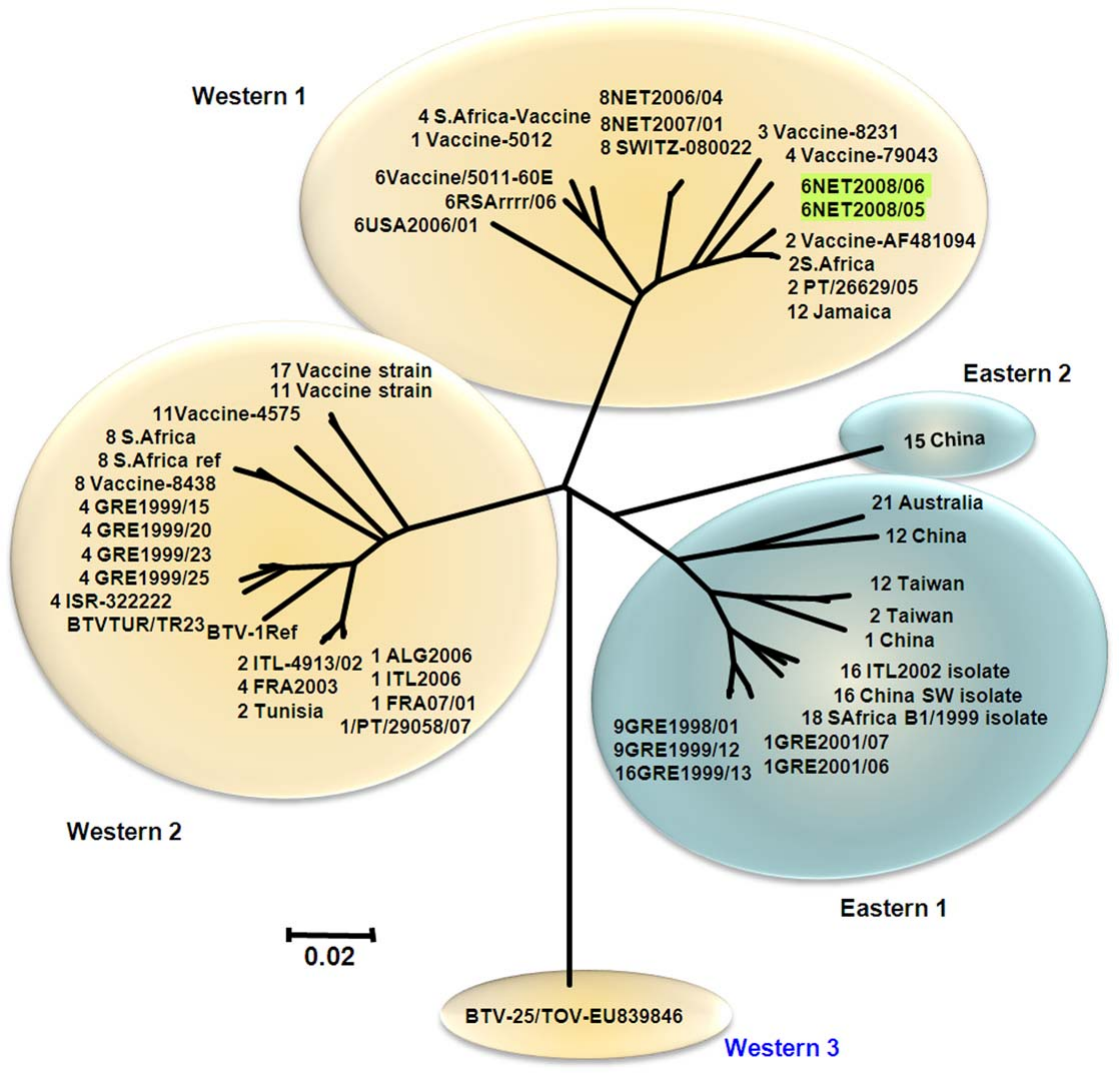
Neighbour-joining tree showing relationships between Seg-10 from BTV-6 NET2008/05 with multiple other BTV-6 isolates and other BTV strains of different serotypes from around the world.

Seg-10 of NET2008/05 is 822 bp long and codes for two small, related non-structural proteins, NS3 (229 aa) and NS3a (216 aa) (Table 2). Seg-10/NS3 sequences can be divided into five groups, with >75.9/81.2% nt/aa conservation overall (Figure 3 and 7). These include three major groups, two from western and one from eastern origins containing the majority of Seg-10 sequences, as well as minor eastern and western groups each containing sequences from only a single isolate (type 15 from China and BTV-25 [TOV] respectively).

The majority of the western viruses (western groups 1 and 2) showed 80.0 to 97.4%/90.4 to 99.6% nt/aa identity to eastern viruses (eastern groups 1 and 2). However ‘western group 3’ represented by the newly discovered BTV-25 (TOV), showed a maximum of 79.1 to 79.3 nt and 83.4% aa identity with the eastern and western isolates respectively (Figure 3 and 7).

Seg-10 from European isolates of BTV-1, 9 and 16 cluster together in eastern group 1, while European isolates of BTV-1, 2 and 4, and South African reference strain of type 1 cluster within western group 2 along with BTV-8 reference strain, and vaccine strains of BTV-8, 11 and 17. Seg-10 of the South African vaccine strains of BTV-1, 2, 3, 4 and 6, cluster within western group 1 along with BTV-8 NET2006/04 and BTV-2 and 6 reference strains and field strains of BTV-2, 6 and 12 from Portugal, USA and Jamaica. NET2008/05 is also included in this western group 1 and was most closely related (98.4/99.6% nt/aa identity) to the reference (Ac. No. AF481094) and vaccine strains of BTV-2 (Ac. No. AF481094), a field strain of BTV-2 from Portugal (EF434179/PT/26629/05) and another field strain of BTV-12 from Jamaica (Ac. No. AY426595). Seg-10 of NET2008/05 and NET2008/06 was more distantly related (93.6/99.6% nt/aa identity) to the BTV-6 vaccine/reference strain and BTV-6 strain from USA (USA2006/01–93.0/99.6%) in western group 1.

## Discussion

In September 2008, cows on three different farms in the eastern Netherlands two of which had previously received an inactivated BTV-8 vaccine, displayed mild clinical signs of BT (particularly coronitis). A fourth apparently healthy cow that was tested for BTV prior to international transport was also RT-PCR positive. Blood samples from these animals gave positive results for the presence of BTV RNA using RT-PCR targeting Seg-10 (at CVI). Partial sequence-analyses of Seg-10 from these four samples showed significant differences when compared to strains of BTV-1 (17.3%) and BTV-8 (3.3%) from northern Europe. These initial data indicated that the virus represented a new introduction to the region.

Conventional RT-PCR assays, using primers directed against Seg-2 of BTV-1, 2, 4, 8, 9 and 16 failed to amplify any products of an appropriate size, providing the first indication that the virus also represented a serotype not previously detected in Europe. Further RT-PCR assays using primers for the 25 established BTV types (1 to 25) identified the virus as BTV-6, although one of the original blood samples from an unvaccinated animal (Luttenberg) also gave positive results with primers targeting Seg-2 of BTV-8, indicating a mixed infection. This demonstrates that both BTV-6 and BTV-8 were circulating in the region. Real-time RT-PCR assays targeting Seg-2 of BTV-1, 2, 4, 6, 8, 9, 11, 16 and 25 (supplied by LSI) subsequently also identified BTV-6 (CT values 14, 27, and 38) in both the blood sample and the virus isolates from Heeten, demonstrating that this strain of BTV-6 could be detected and identified by these assays.

Neutralisation assays demonstrated the presence of antibodies to both BTV-6 and 8 in serum from the infected animal at Heeten, reflecting infection with BTV-6 as well as infection and/or vaccination with BTV-8.

BTV-6 had previously been isolated from Africa, Middle East, India, Pakistan, USA, Central and South America [87] and therefore exists as both eastern and western strains, although no sequence data for the eastern strains were available for these analyses. The data presented here represent the first complete sequence (segments 1-10) of the northern European strain of BTV-6, as well as BTV-6 field and vaccine strains from South Africa and a BTV-6 field strain from North America (USA2006/01).

Sequence analyses of Seg-2 from the Heeten blood sample (NET2008/06) and isolate (NET2006/05) showed that it clusters in ‘Seg-2 nucleotype C’ (identified by Maan et al. [57], [72]–Figure 4), and revealed 5 nt differences (99.8/99.7% nt/aa identity) from the reference strain of BTV-6 (RSArrrr/06 - isolated in South Africa in 1958: reviewed by Alpar et al. [39]). This not only confirmed the virus type, but also indicated that it belongs to the major ‘western’ topotype, and groups with other sub-Saharan African strains of BTV-6. Further comparisons of Seg-2/VP2, showed that it is identical to the South African live attenuated vaccine strain of BTV-6, providing the first indication that it was in fact derived from this source.

Seg-2 and Seg-6 of TOV [65] were also included in these analyses, showing levels of variation that are consistent with its inclusion as BTV-25, as a representative of a distinct eleventh nucleotype of Seg-2 (nucleotype K - Figure 4) and an eighth nucleotype of Seg-6 (nucleotype H–Figure 5). Comparisons of VP3 from the Toggenburg orbivirus isolate [65] showed 88.0 to 89.5% aa identity with other BTV strains. This is significantly higher than the 77.3 to 77.9% identity which is shares with isolates of epizootic haemorrhagic disease virus (EHDV) [88], the most closely related of the other *Orbivirus* species to BTV [1]. BTV and EHDV themselves also show only 78.5 to 81.1% aa identity in VP3. These values compare with 91%, the lowest level of identity previously detected in VP3 within a single *Orbivirus* species [89]. If TOV is to be considered as a new strain/serotype of BTV, this pushes down the level of identity for VP3 within a single species, although this is perhaps inevitable as more distinct and diverse isolates are analysed and compared. Further confirmation that TOV is a member of the species *Bluetongue virus* could be generated by a demonstration that it can exchange/reassort genome segments with other strains of BTV [1]. However, attempts to adapt the virus to cell culture have so far been unsuccessful [65], making these experiments more difficult.

Phylogenetic analyses of the other genome segments from NET2008/05 showed that most were very similar or identical to the South African live-vaccine strain of BTV-6 (99.7–100% nt/aa identity). They also contained significantly higher numbers of nucleotide changes when compared to sequences from field strains of other serotypes already circulating in Europe, or the other live-vaccine strains that had been used in the Mediterranean region. Subsequent data for BTV-6 isolated in Germany during 2008 (GER2008/05) showed small numbers of nucleotide changes in several of genome segments, when compared to NET2008/05, but confirmed that the different isolates were all very closely related representing a single virus lineage. All of the genome segments of NET2008/05 group with those of other ‘western’ viruses, belonging to sub-Saharan African lineages. However, Seg-10 of NET2008/05 was less closely related than the other segments to that of the BTV-6 vaccine strain (93.6/99.6% nt/aa identity) and was clearly distinct from previously published data for other field strains from Europe or elsewhere. Seg-10 of NET2008/05 was most closely related to that of the South African vaccine and reference strains of BTV-2, showing 98.4/99.6% nt/aa sequence identity. This indicates that they share a recent common ancestry and suggests that the Netherlands virus acquired Seg-10 by reassortment, possibly with another BTV vaccine strain. Although it is possible that this segment was acquired during co-infection by BTV-2 and BTV-6 vaccines, the level of variation observed (1.6%) suggests that this would have happened several cycles of replication before the virus arrived in northern Europe.

When BTV-6 was detected in the Netherlands during October 2008, it represented a further new serotype to Europe. Its African and vaccine strain origins are indicated by the phylogenetic analyses reported here. Recent studies also detected Seg-2 of the South African BTV-11 vaccine strain in the same part of northern Europe (in Belgium) during early 2009 [90]. Together with the arrival of BTV-8 in the Maastricht region during August 2006, this indicates that there is an effective route for the introduction of these viruses (none of which had previously been detected in Europe) to this part of northern Europe, although details of the mechanisms involved remain a matter for speculation.

The BTV-6 vaccine has previously been used are in Israel and South Africa itself. In each case it was used as part of a multivalent vaccine. In Israel this involved BTV-2, -4, -6, -10 and -16, while in South Africa the ‘Bottle-A’ vaccine contains BTV-1, -4, -6, -12 and -14. It has been suggested that the virus could have arrived in the region as the result of illegal use of these live attenuated BTV vaccines (‘Bottle-A’ of the south African BTV vaccine contains BTV-1, a strain of current concern in many regions of Europe). However, the absence of antibodies to multiple other serotypes in the antiserum from Heeten indicates that this animal at least had not received either of the multivalent vaccine preparations produced by Onderstepoort Biological Products (OBP) in South Africa.

Like most of the other genome segments (Seg-1 to 9), Seg-7/VP7 of NET2008/04 (KC isolate) and NET2008/05 (KC1/BHK1 isolate) showed 99.7-100% nt/aa identity to the South African BTV-6 live-vaccine strains. Similar results were also obtained with the virus after isolation in embryonated chicken eggs and passage in BHK-21 cells (at CVI). However, Seg-7 from the Heeten blood sample from (NET2008/06) generated a consensus Seg-7/VP7 sequence that was identical to Seg-7/VP7 of BTV-8 from the Netherlands 2006 (NET2006/04) and 2007 (NET2007/01), but only showed ∼80.5% nt identity to the BTV-6 isolates from the same sample (Figure 6). This suggests that the original blood contained two different versions of Seg-7 (consistent with the circulation of both BTV-6 and BTV-8 in the region), only one of which (from the tissue-culture adapted BTV-6 vaccine strain) was selected during isolation of the virus in cell-culture. It is possible that this reflects some involvement of the selected version of VP7 in the infection mechanism and consequently in adaptation of the virus to cell culture. However, the predominance of the BTV-8 Seg-7 in the original Heeten blood sample (NET2008/06) suggests that the virus was in the process of reassorting with the northern field strain of BTV-8 that was widespread in northern Europe.

We have considered the possibility that Seg-7 from the Netherlands strain of BTV-8 could have been detected in the blood sample from Heeten, as a result of laboratory contamination. However, our laboratory works to QA standards (ISO9001) and although the sequencing of Seg-7 was repeated three times, using freshly extracted RNA on each occasion, consistent results were obtained. Other samples extracted and tested at the same time did not contain BTV-8 Seg-7 and no other BTV-8 sequences (from other segments) were detected in the blood sample, collectively excluding the possibility of accidental contamination with BTV-8 Seg-7, or with BTV-8 virus.

In order to exchange genome segments these two ‘parental’ viruses (BTV-6 and BTV-8) must co-infect the same cells within an individual mammalian host or insect vector, and this appears most likely to have occurred after BTV-6 was introduced into northern Europe. Analyses of German 2008 samples by real-time RT-PCR (supplied by LSI) identified several animals that were infected by both BTV-6 and 8 (GER2008/01 and GER2008/02). These samples were also shown to contain a mix of the two different versions of Seg-7 detected from the Heeten blood and virus isolate.

It may be significant that evidence for reassortment was only detected in Seg7/VP7 and Seg-10/NS3/3a of the BTV-6 strain from northern Europe. VP7 is known to be involved in binding of BTV core particles to the cell surface membrane proteins of vector *Culicoides* sp and may play an important role in BTV infection of the insect vector [66], [91]. In contrast NS3/3a are involved in the release of virus particles from infected insect cells [67], [68], [92] and together with VP7 (and possibly the other outer coat protein VP2 and VP5 involved in cell attachment) may influence the dissemination of the virus within the individual insect. It has been suggested that variations in these two genome segments and proteins (Seg-7/VP7 and Seg10/NS3/3a) may reflect transmission of BTV by different vector species or populations in different geographic locations [35]. The acquisition of different versions of Seg-7 and Seg-10 by the vaccine strain of BTV-6 could therefore reflect adaptation to transmission by the vector population within the ecosystem that exists in northern Europe. However, further study will be needed to show if there are differences in the infection rate in north European *Culicoides* sp, or the transmission efficiency for these viruses containing the different versions of Seg-7/VP7.

The provision of a full genome sequence for the northern European field strain of BTV-6 will make it possible to track any further changes or reassortment events that occur if the virus continues to persist in the region.

## Supporting Information

Table S1 Accession numbers of BTV genome segments (Seg-7 and Seg-10) used in these phylogenetic analyses, in addition to those described previously by Maan et al [35], [57], [72].
(0.06 MB DOC)
