# Supplementary material for: Full Genome Characterisation of Bluetongue Virus Serotype 6 from the Netherlands 2008 and Comparison to Other Field and Vaccine Strains
Source: PLoS One. 2010 Apr 23;5(4):e10323. doi: 10.1371/journal.pone.0010323 (PMC2859060; doi:10.1371/journal.pone.0010323)
Supplement: Table S1 — Accession numbers of BTV genome segments (Seg-7 and Seg-10) used in these phylogenetic analyses, in addition to those described previously by Maan et al [35], [57], [72]. (0.06 MB DOC) [file pone.0010323.s001.doc]

**Table S1. Accession numbers of BTV genome segments (Seg-7 and Seg-10) used in these phylogenetic analyses, in addition to**

**those described previously by Maan et al [35, 57, 72].**

| **IAH Reference collection number or Year of isolation or strain designation** | **Country of origin** | **Seg-7** | **Seg-10** |
| --- | --- | --- | --- |
| 1 Algeria 2006 | Algeria |  | EF533883 |
| 15 Australia | Australia | L11723 |  |
| 15 China | China |  | AF135228 |
| 16 China SW isolate | China |  | AF135229 |
| BTV-? China (Yunnan China) | China | AY386682 |  |
| 3 Costa Rico (2058 isolate) | Costa Rico | AF188651 |  |
| 3 Costa Rico (2354 isolate) | Costa Rico | AF188655 |  |
| 4 Dominican Republic (2227 isolate) | Dominican Republic | AF188667 |  |
| 8 Dominican Republic (2215 isolate) | Dominican Republic | AF188671 |  |
| 3 El Salvador (2270 isolate) | El Salvador | AF188645 |  |
| 1 France (FRA07/01) | France |  | FJ437561 |
| 4 France 2003 | France |  | AY857503 |
| 4 Greece 1999 | Greece | AY841352 |  |
| 4 Greece 2000 | Greece | AY841351 |  |
| 3 Guatemala (2350 isolate) | Guatemala | AF188663 |  |
| 17 Guatemala (240 isolate) | Guatemala | AF188664 |  |
| 3 Honduras (2154 isolate) | Honduras | AF188646 |  |
| 4 Israel (ISR-322222) | Israel |  | AY775157 |
| 1 Italy 2006 | Italy |  | EF533882 |
| 2 Italy (ITL-4913/02) | Italy |  | AY775153 |
| 12 Jamaica | Jamaica |  | AY426595 |
| 3 la Reunion | la Reunion | AY485667 |  |
| 3 Panama (2343 isolate) | Panama | AF188658 |  |
| 3 Panama (2364 isolate) | Panama | AF188654 |  |
| 1 Portugal (PT29058/07) | Portugal |  | EU498676 |
| 2 Portugal (PT26629/05) | Portugal |  | EF434179 |
| 2 Portugal (Grandola/05) | Portugal | EF434175 |  |
| 17 Puerto Rico (300 isolate) | Puerto Rico | AF188666 |  |
| 17 Puerto Rico (285 isolate) | Puerto Rico | AF188665 |  |
| 2 South Africa | South Africa |  | AF481094 |
| 2 South Africa (557 isolate) | South Africa | AF188672 |  |
| 2 South Africa (Vaccine) | South Africa | AF481095 |  |
| 3 South Africa (RSArrrr/03) | South Africa | AF188649 |  |
| 4 South Africa vaccine | South Africa |  | AY857506 |
| 7 South Africa (RSArrrr/07) | South Africa | DQ465026 |  |
| 8 South Africa | South Africa |  | EU450663 |
| 8 South Africa | South Africa |  | AF512924 |
| 15 South Africa (RSArrrr/15) | South Africa | DQ465027 |  |
| 18 South Africa B1/1999 | South Africa |  | AF512914 |
| 19 South Africa (RSArrrr/19) | South Africa | DQ465028 |  |
| 8 Switzerland (SWITZ-080022) | Switzerland |  | EU450661 |
| BTV-? Turkey (TR23) | Turkey |  | EF554855 |
| 3 Tobago (2045 isolate) | Tobago | AF188652 |  |
| 3 Trinidad (2165 isolate) | Trinidad | AF188647 |  |
